# Three-year safety observation of subcutaneous administration of epoetin-zeta in patients with chronic renal anemia: Results from PASCO II study 

**DOI:** 10.5414/CN110825

**Published:** 2023-03-05

**Authors:** Stavros Patsialas, Heather Fowler, Ruffy Guilatco, Stephanie Salts, Feng Richard Xia, Sonja Gomez Perez, Andreas Iwanowitsch, Matthias Kohnle

**Affiliations:** 1 Nephroiasis Dialysis Unit, Thessaloniki, Greece,; 2 Pfizer Ltd, Walton Oaks, Surrey, UK,; 3 Pfizer Inc., Makati City, Philippines,; 4 Pfizer Inc., San Diego, CA,; 5 Pfizer Inc., New York, NY, USA,; 6 STADA Arzneimittel AG, Bad Vilbel, and; 7 Nephrocare Mettmann GmbH, Mettmann, Germany

**Keywords:** renal anemia, epoetin, pure red cell aplasia (PRCA), biosimilar

## Abstract

Epoetin has been used to treat patients with renal anemia since 1988. Anti-erythropoietin antibody-mediated pure red cell aplasia (PRCA) has been associated with epoetin usage, and a PRCA incidence of 4.5 per 10,000 patient-years was observed for epoetin-α (Eprex) in 2002. The PASCO II study (post-authorization safety cohort observation of Retacrit and Silapo (epoetin-ζ) administered subcutaneously for the treatment of renal anemia) followed 6,346 patients (4,501 Retacrit (group R); 1,845 Silapo (group S)) for up to 3 years of subcutaneous treatment with the biosimilar epoetin-ζ. One PRCA in 1 (0.02%) patient in group R who tested positive for neutralizing antibodies was reported. Overall, 527 adverse events of special interest (AESI) including PRCA occurred in 418 (6.60%) patients, lack of efficacy occurred in 34 (0.54%), and thromboembolic events in 389 (6.14%) patients. 41 adverse drug reactions other than AESIs were reported in 28 (0.44%) patients. The exposure-adjusted incident rate of PRCA was 0.84 per 10,000 patient-years. This real-world study showed that among patients with renal anemia receiving subcutaneous administration of the biosimilar product epoetin-ζ, the incidence rate of PRCA was substantially below the risk observed in 2002 for Eprex and that there was no immunogenicity concern or other new safety concern.

ClinicalTrials.gov registration: NCT01543477

## Introduction 

Recombinant human erythropoietin (epoetin) has been used to treat patients with renal anemia since 1988. As a serious adverse reaction, anti-erythropoietin antibody-mediated pure red cell aplasia (PRCA) has been associated with epoetin usage [1]. Since 1998, an increase in cases of epoetin-associated PRCA had been reported in patients receiving erythropoiesis-stimulating agent (ESA) therapy. More than 90% of the PRCA cases were in patients treated with epoetin-α (Eprex; Johnson & Johnson, New Brunswick, NJ, USA), and almost all patients had chronic kidney disease and received epoetin via subcutaneous (SC) injection [2, 3]. Further studies showed that between 1998 and 2003, the exposure-adjusted incidence rates of PRCA in patients with chronic kidney disease were 0.2 – 4.5 per 10,000 patient-years for epoetin-α (Eprex), 0.14 – 0.2 per 10,000 patient-years for epoetin-β (Neorecormon; Roche Registration GmbH, Grenzach-Wyhlen, Germany), and 0.06 – 0.2 per 10,000 patient-years for epoetin-α (Epogen; Amgen, Thousand Oaks, CA, USA or Procrit; Janssen Products, LP, South Raritan, NJ, USA) [3, 4, 5, 6]; and the increased risk of epoetin-associated PRCA was found to be related to SC administration [4]. Following reformulation of Eprex and the subsequent pharmacovigilance efforts and safety guidance, epoetin-associated PRCA became a rare class-related toxicity for patients with chronic renal failure receiving extended treatment periods of SC epoetins, with an incidence rate of 0.02 – 0.03 per 10,000 patient-years [6]. Patients with renal anemia were considered as the population at-risk, and SC administration of epoetin was identified as a risk factor [6]. 

Biosimilar epoetins have been approved as effective alternatives to the reference medicines in Europe for 14 years [7]. Epoetin-ζ (Retacrit^1^; Pfizer Europe MA EEIG, Brussels, Belgium, and Silapo; STADA Arzneimittel AG, Bad Vilbel, Germany; the drug substance for both is manufactured by Norbitech GmbH, Uetersen, Germany) is a biosimilar to the innovator product of epoetin-α. It is approved in the intravenous (IV) and SC administrations for the treatment of symptomatic anemia associated with chronic renal failure and other clinical conditions that require ESA therapy [8, 9]. Post-authorization observational studies have been carried out to obtain real-world data to further assess the safety profiles of epoetin-ζ compared with the historical data of the reference product, epoetin-α, including the risk of PRCA. 

^1^Retacrit was approved by the U.S. Food and Drug Administration in the U.S. in 2018, the active substance was named epoetin alfa-epbx. 

The previous PASCO I study (post-authorization safety cohort observation of Retacrit/Silapo (epoetin-ζ) administered intravenously for the treatment of renal anemia) was carried out in 4 European countries (Germany, Spain, Italy, and the United Kingdom) between September 2008 and August 2011 [10]. This non-interventional, non-comparative, multicenter, prospective safety cohort study included 1,634 patients with renal anemia who received IV epoetin-ζ and were observed for up to 1 year. Results of PASCO I showed that the safety profiles of epoetin-ζ were similar when compared with other epoetin products used in countries in the European Union (EU), and no PRCA events were reported [10]. 

To further assess the safety profile of epoetin-ζ, including the risk of epoetin-associated PRCA, when using a different route of administration (SC rather than IV injection), the PASCO II study (post-authorization safety cohort observation of Retacrit and Silapo (epoetin-ζ) administered subcutaneously for the treatment of renal anemia) was designed. The PASCO II study planned to have a prospective cohort of > 6,000 patients, each to be followed for up to 3 years of treatment with SC epoetin-ζ. It aimed to collect long-term safety data of epoetin-ζ and to detect cases of epoetin-associated PRCA to determine if the incidence rate of PRCA with SC epoetin-ζ treatment in renal anemic patients was substantially below the incidence of 4.5 per 10,000 patient-years observed for epoetin-α (Eprex) in 2002 [6]. 

## Materials and methods 

### Study design 

PASCO II was a non-interventional, longitudinal, multicenter, prospective cohort study of patients with renal anemia treated with SC epoetin-ζ (Retacrit or Silapo). It included two separate but matching protocols. Protocol EPOE-09-11 (C1111006) investigated treatment with Retacrit (group R); it was conducted by Hospira and enrolled patients in several European countries. Protocol PMS-830-09-0082 investigated treatment with Silapo (group S); it was conducted by STADA and enrolled patients solely in Germany. 

Eligible patients were those who started on treatment with SC epoetin-ζ for renal anemia according to the current Summary of Product Characteristics (SmPC [8, 9]). Enrolled patients needed to provide written informed consent and be available for 3 years of observation. Patients with any contraindications per the current SmPCs were excluded. Observations were conducted at nephrologists’ practices and dialysis centers treating patients with renal disease. 

PASCO II was approved by the European Medicines Agency (EMA) Committee for Medicinal Products for Human Use (​CHMP) as a post-authorization safety study. The final protocol, any amendments, and informed consent documentation used by the sites were reviewed and approved by (or notified to) the Independent Ethics Committees in accordance with local requirements. The study was conducted in accordance with the Declaration of Helsinki and applicable local regulatory requirements and laws. 

### Assessments 

The primary endpoint was to determine the incidence rates of adverse events of special interest (AESIs): PRCA, neutralizing antibodies, lack of efficacy, and thromboembolic events including cerebrovascular events (e.g., cerebrovascular accident, cerebral infarction, cerebral hemorrhage, and transient ischemic attack), deep vein thrombosis, myocardial infarction, and pulmonary embolism. The secondary endpoints were the incidence rates of adverse drug reactions (ADRs) and information on pregnancy/lactation exposure and long-term use. 

AESIs and ADRs were the only clinical events required to be reported for this observational study. In cases where lack of efficacy or hemoglobin decreases (but no event of PRCA) were reported, the cause of the event was assessed to confirm or rule out whether the reported events represented clinical signs or symptoms of potential immunogenicity or PRCA. For suspected PRCA cases, a confirmatory test for anti-erythropoietin antibodies of a recent blood sample was offered. 

Demographics; medical history, including selected risk factors, diagnosis leading to renal failure and dialysis information; details on epoetin-ζ treatment; and premature treatment termination were also assessed. 

### Statistical methods 

PASCO II was designed primarily to verify that no immunogenicity concern arises from the subcutaneous use of epoetin-ζ. Since the highest exposure-adjusted incidence rate of epoetin-associated PRCA was 4.5 per 10,000 patient-years in 2002 [6], 6,700 patients were planned to be enrolled in this observational study and followed for up to 3 years of treatment with SC epoetin-ζ. This planned sample size would be sufficient to detect cases of epoetin-associated PRCA to demonstrate that the incidence rate under treatment with SC epoetin-ζ is substantially below the highest incidence rate observed. 

Since the study start in 2010, the observed incidence rate of PRCA over a period of nearly 9 years in this study was substantially below the rate observed in 2002 for Eprex, and the probability of an increase in the estimated incidence rate of PRCA was low if additional patients were observed. Following consultation with the CHMP Pharmacovigilance Risk Assessment Committee (PRAC), it was agreed to reduce the total sample size to a minimum of 6,206 patients (total number of patients enrolled at the time of the consultation). The approved sample size reduction was documented in a protocol amendment 9.5 years after the study had started. As a result, the observation of ongoing patients in the STADA study was to end at the time the last patient in the Hospira study completed their 3-year follow-up, which enabled the Hospira and STADA data to be combined and jointly analyzed. 

The PASCO II study data set was derived from two matched observational, non-comparative studies, and any analyses carried out using the pooled data was descriptive and exploratory. The enrolled set was defined as all patients who provided informed consent and were enrolled in the study. It was used to summarize patient disposition, medical history, and demographic and baseline characteristics. The safety set was defined as all patients who received ≥ 1 dose of epoetin-ζ during the observation period. 

Continuous variables are presented using descriptive summary statistics: number of observations, arithmetic mean, standard deviation, median, minimum, and maximum values. Categorical variables are presented using frequency counts and percentages. Missing data were not imputed. 

A sensitivity analysis was performed, which excluded data collected > 38 months after the date of informed consent. 

## Results 

### Patient disposition and demographics 

**Figure 1:**
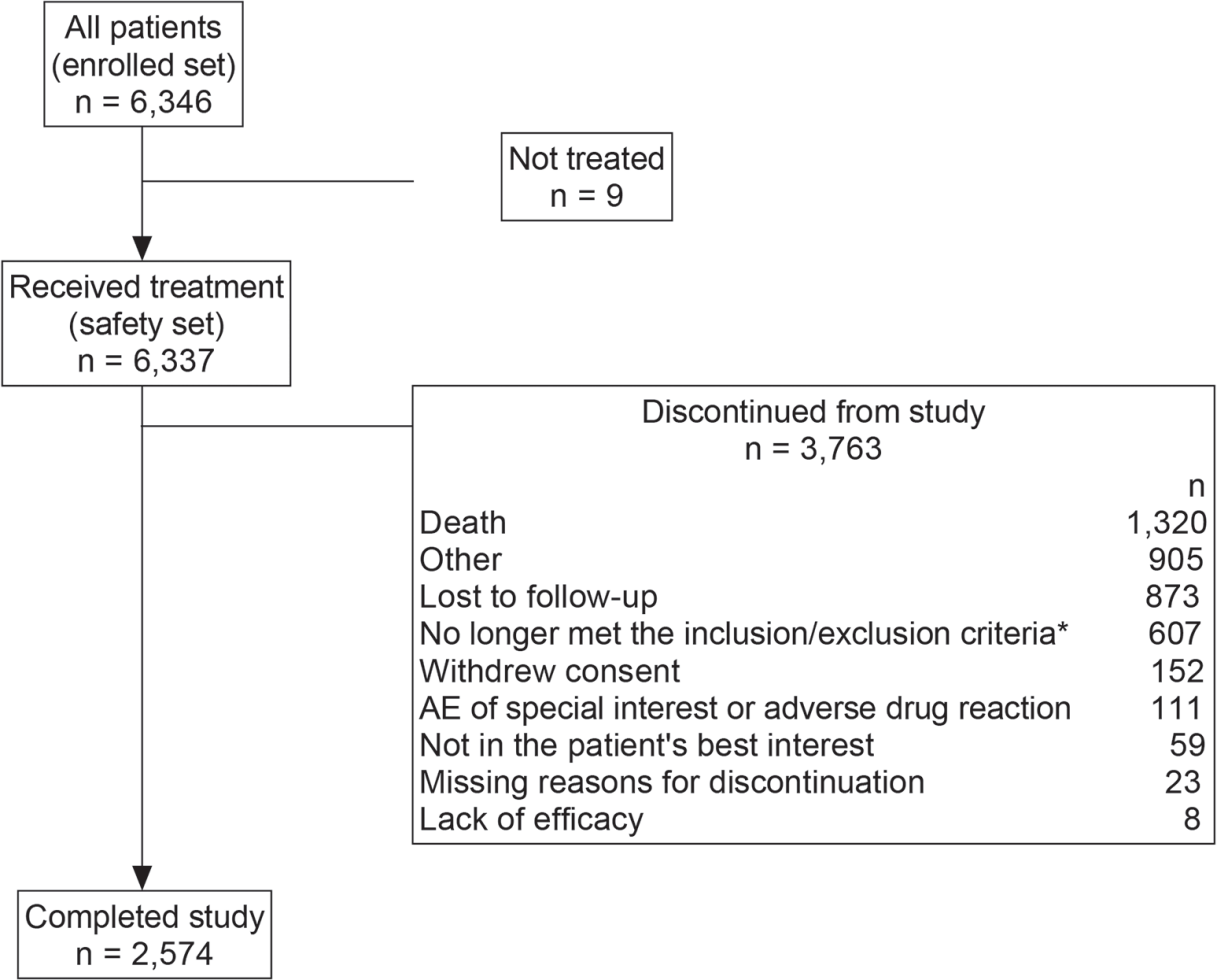
Patient disposition. *No longer met inclusion/exclusion criteria included patients who switched to another erythropoiesis-stimulating agent treatment. AE = adverse event.

PASCO II was conducted between July 2010 and May 2020 in 12 European countries (Bulgaria, Croatia, Denmark, Finland, France, Germany, Greece, Ireland, Italy, Spain, Sweden, and the United Kingdom). A total of 6,346 patients (group R: n = 4,501; group S: n = 1,845) were enrolled. Of these, 6,337 patients (group R: n = 4,496, group S: n = 1,841) received epoetin-ζ and were included in the safety set (Figure 1). Overall, 3,763 (59.3%) patients discontinued from the study prior to completing the 3-year observation period. The most common reason for discontinuation was death (n = 1,320 (20.8%)). 

Demographic characteristics were comparable across both studies. A numerically higher proportion of male (55.5%) than female (44.5%) patients were included. Most patients were White (98.2%), with a mean (standard deviation (SD)) age of 71.2 (13.8) years in group R and 70.7 (14.0) years in group S. Patients had mean (SD) hemoglobin values of 10.5 (1.4) g/dL (range 6.0 – 19.0 g/dL) and mean hematocrit (proportion of 1.0) values of 0.32 (SD 0.04; range 0.19 – 0.53) at baseline (Table 1). 

### Medical history 

Out of the 6,343 patients with a diagnosis leading to renal failure at baseline, the majority (4,788 (75.5%)) were diagnosed with renal and urinary disorders, the most common of which were hypertensive nephropathy (1,949 (30.7%)), diabetic nephropathy (1,610 (25.4%)), and glomerulonephritis (696 (11.0%)). Overall, 2,274 (35.8%) patients (1,236 (27.5%) patients from group R, 1,038 (56.3%) patients from group S) had a history of dialysis prior to study entry. The mean frequency of dialysis sessions per week was 3.1 (SD 0.8; range 0 – 7 sessions). The most frequent medical risk factors (≥ 20% of patients) assessed at baseline were hypertension 5,409 (85.2%), type 2 diabetes mellitus 2,564 (40.4%), hyperlipidemia 1,955 (30.8%), and coronary artery disease 1,830 (28.8%) (Table 1). 

62% of patients had received previous treatment for renal anemia. The most common prior ESA treatment was epoetin-ζ. 

### Outcomes 

AESIs were reported for 418 (6.6%) patients and ADRs other than AESIs for 28 (0.4%) patients (Table 2) (Supplementary Table 1). Out of the 527 AESIs reported, 516 events in 409 (6.5%) patients were considered serious (Supplemental Tables 1, 2). 

One event of PRCA was reported on day 250 in 1 (0.02%) patient in group R who had positive test results for neutralizing antibodies against erythropoietin. This PRCA event was considered serious and related to epoetin-ζ. The patient was withdrawn from the study due to this PRCA event and due to lack of efficacy (lack of efficacy was reported as a separate serious AESI of the PASCO II study). The patient later recovered from the event of PRCA, while the event of lack of efficacy was unresolved. The incidence of PRCA derived from the life-table analysis with 2-monthly intervals was 0.000202 at 8 – 10 months, and 0 through the remainder of the observation period. The incidence of neutralizing antibodies derived from the life-table analysis with 2-month intervals was 0.000213 at 10 – 12 months, and 0 through the rest of the observation period. 

Overall, the most common AESIs were thromboembolic events, reported for 389 (6.1%) patients; cardiac disorders (163 (2.6%) patients), nervous system disorders (114 (1.8%) patients), and vascular disorders (83 (1.3%) patients) were the most common system organ classes. 

Myocardial infarction was the most frequent single thromboembolic event (by preferred term) reported for 114 (1.8%) patients (Table 2). Most patients for whom thromboembolic events were reported had ≥ 1 serious event (387/389 patients). All events of myocardial infarction were considered serious. The incidence of thromboembolic events derived from the life-table analysis with 2-month intervals ranged from 0.00895 at the beginning of the study (0 – 2 months) to 0.00121 at the end of the observation period (34 – 36 months). No obvious pattern in probability over time was observed. 

Lack of efficacy was reported for 34 (0.5%) patients; it was considered as a serious AESI in 26 (0.4%) patients (Table 2, Supplementary Table 2). Of these 34 patients, 10 recovered with 8 switched to another ESA, 1 had been switched to another ESA and was recovering, 12 did not recover with 3 switched to another ESA, and the outcomes were unknown for 11 patients. The incidence of lack of efficacy derived from the life-table analysis with 2-month intervals ranged from 0.00163 at the beginning of the study (0 – 2 months) to 0 at the end of the observation period (34 – 36 months). No obvious pattern in probability over time was observed. 

A total of 41 ADRs other than AESIs were reported for 28 (0.4%) patients. Five patients in group S had both AESIs and other ADRs reported (Supplementary Table 1, Supplementary Table 3). The most frequent single ADR was hemoglobin decrease (by preferred term), which was reported for 5 patients (all in group S). The majority of other ADRs were reported in 1 patient each. Other than decreased hemoglobin, malaise, headache, and pruritus were reported in 3 patients each, and nausea, dizziness, and allergic dermatitis were reported in 2 patients each. 13 ADRs in 11 (0.2%) patients were considered serious. All events of decreased hemoglobin were considered serious. Two ADRs were fatal (1 decreased hemoglobin, 1 gangrene) and both were in group S. The incidence of ADRs derived from the life-table analysis with 2-month intervals ranged from 0.00277 at the beginning of the study (0 – 2 months) to 0 at the end of the observation period (34 – 36 months). No obvious pattern in probability over time was observed. 

Only 1 patient developed PRCA and had positive results for neutralizing antibodies during the study observation, which translated to an exposure-adjusted incidence rate of 0.84 (95% confidence interval (CI): 0.04 – 5.49) per 10,000 patient-years. For lack of efficacy, thromboembolic events, and ADRs other than AESIs, the exposure-adjusted incidence rate was 0.29 (95% CI: 0.20 – 0.40), 3.37 (95% CI: 3.05 – 3.72), and 0.24 (95% CI: 0.16 – 0.34) per 100 patient-years, respectively (Table 3). 

### Other analysis 

Following the implementation of the protocol amendment approved by the CHMP PRAC of EMA to reduce the sample size, some patients in the STADA study (group S) discontinued prior to completing their 3-year follow-up to coincide with the completion of 3-year follow-up for the last patient enrolled in the Hospira study. The last patients’ last visits to the Hospira and the STADA study were April 29, 2020 and May 29, 2020, respectively. Therefore, the median exposure in group R was 725.5 days and 592.0 days in group S, whereas the overall mean (SD) exposure to epoetin-ζ was 666.4 (392.5) days (range 1 – 1,437 days). For the whole study population, 84.0% of patients received treatment with epoetin-ζ for up to 36 months in total, equating to 8,703.0 patient-years (Supplemental Table 4). No exposure to epoetin-ζ during pregnancy or lactation during the observation period of this study was reported. 

Overall, 144 patients (group R: n = 71; group S: n = 73) were treated and observed for longer than 38 months. The mean (SD) duration of exposure beyond 38 months was 50.2 (55.0) days (range 1 – 282 days). Two patients (in group S) had AESIs while receiving treatment beyond the 38-month period. Sensitivity analyses showed that the impact on the exposure-adjusted incidence rate of primary and secondary endpoints was negligible. 

## Discussion 

PASCO II was a large-scale, long-term, real-world observational study that collected up to 3 years of safety data of SC epoetin-ζ in the treatment of patients with renal anemia. A single event of epoetin-associated PRCA was observed in 1 patient out of the 6,337 patients included in the safety analysis set of this study. The details of this case of PRCA have been reported previously [11]. The exposure-adjusted incidence rate of PRCA was 0.84 per 10,000 patient-years, which was substantially lower than the incidence rate of 4.5 per 10,000 patient-years observed for Eprex (epoetin-α, Johnson & Johnson, USA) in 2002 [6]. In addition, the overall percentage of patients with other AESIs was low; lack of efficacy was observed for 0.54% of patients. For thromboembolic events, the observed frequency in PASCO II (6.14%) was consistent with the frequency category in the current EU SmPC, which is “common” (> 1% to < 10%) [8, 9]. The PASCO II study was designed primarily to verify that no immunogenicity concern arises from the subcutaneous use of epoetin-ζ. Due to the large sample size, this observation was also helpful in providing further information about the incidence of thromboembolic events in patients with renal anemia treated with epoetin-ζ. 

Epoetins have been used both intravenously and subcutaneously; SC administration is more common compared with the IV route, as the SC route uses a lower dose of epoetin with better outcomes at lower cost [12, 13, 14, 15]. After the peak incidence rate of PRCA associated with epoetin-α treatment in 2002 [6], few cases of PRCA caused by biosimilar epoetin products have been reported, these included 2 cases for HX575 (binocrit; Sandoz GmbH, Kundl, Austria) in 2012 (1 confirmed, 1 possible) [16], 1 case for epoetin-θ/epoetin-β/darbepoetin-α in 2013 [17], and this case for epoetin-ζ during the study period of PASCO II. All of these PRCA cases occurred in patients with renal anemia who received SC epoetins [11, 16, 17]. The safety profiles of these biosimilars were overall comparable with the epoetin reference products [18]. 

Considering that this non-interventional study was conducted in a broad real-world study population of patients with renal anemia in more than 10 European countries and with minimal, less restrictive inclusion criteria, the study data can be generalized. However, this study has some limitations. This is a non-comparative observational study that lacks a randomized control. The reduced study sample size translated to slightly wider CIs around the estimated incidence rates. As some patients were observed in the study for longer than the planned 3 years, a greater number of safety events were reported. Further, infrequent study visits may have resulted in decreased reporting of safety events as patients participating in this study may have forgotten that the events had occurred since the last visit. Lastly, the study was conducted over a 10-year period so there were inevitable changes in investigators, site staff, and sponsor study team members as well as closures of some study sites leading to unaddressed queries. 

In conclusion, PASCO II demonstrated that the safety profile of SC epoetin-ζ (Retacrit or Silapo) in patients with renal anemia was comparable to that of the epoetin reference product, and no new safety concerns were identified. Based on the observed incidence rate in this study, there is no immunogenicity concern of anti-erythropoietin antibody-mediated PRCA. 

## Availability of data 

Upon request, and subject to review, Pfizer will provide the data that supports the findings of this study. Subject to certain criteria, conditions, and exceptions, Pfizer may also provide access to the related individual de-identified participant data. See https://www.pfizer.com/science/clinical-trials/trial-data-and-results for more information. 

## Acknowledgment 

The authors thank the participating patients and their families/caregivers, participating physicians, and the investigators, co-investigators, and site staff who contributed to this study. Medical writing support was provided by Shuang Li, PhD, of Engage Scientific Solutions, and was funded by Pfizer. 

## Funding 

This observation study was sponsored by Hospira Inc., a Pfizer company, and by STADA Arzneimittel AG. 

## Conflict of interest 

Matthias Kohnle and Stavros Patsialas were principal investigators of the study PASCO II. Ruffy Guilatco, Stephanie Salts, and Feng Richard Xia are employees of Pfizer with stock and/or stock options. Heather Fowler is a consultant for Pfizer Inc. Sonja Gomez Perez and Andreas Iwanowitsch are employees of STADA Arzneimittel AG. 

**Table 1.** Demographic and baseline characteristics.

	Total (n = 6,346)
Age of group R, n	4,500
Mean (SD), years	71.2 (13.8)
Median (range), years	75.0 (0 – 99)
Age of group S, n	1,842
Mean (SD), years	70.7 (14.0)
Median (range), years	74.0 (18 – 97)
Sex	
Male	3,523 (55.5)
Female	2,821 (44.5)
Missing	2 (0.0)
Race*	
Asian	24 (0.4)
Black	10 (0.2)
White	6,233 (98.2)
Other	10 (0.2)
Missing	69 (1.1)
Smoking status	1,636 (25.8)
Ex-tobacco user	1,146 (18.1)
Tobacco user	490 (7.7)
Hemoglobin, n	6,329
Mean (SD), g/dL	10.51 (1.37)
Median (range), g/dL	10.50 (5.96 – 19.00)
Hematocrit, n	6,280
Mean (SD), proportion of 1.0	0.32 (0.04)
Median (range), proportion of 1.0	0.32 (0.19 – 0.53)
Heart rate^†^, n	5,709
Mean (SD), bpm	72.6 (10.1)
Median (range), bpm	72.0 (0 – 142)
Medical conditions^‡^	
Atrial fibrillation	966 (15.2)
Cardiac failure chronic	1,247 (19.7)
Coronary artery disease	1,830 (28.8)
Myocardial infarction	732 (11.5)
Hyperlipidemia	1,955 (30.8)
Type 2 diabetes mellitus	2,564 (40.4)
Neoplasm malignant	322 (5.1)
Cerebrovascular accident	415 (6.5)
Cerebrovascular disorder	362 (5.7)
Embolism venous	188 (3.0)
Hypertension	5,409 (85.2)
Peripheral arterial occlusive disease	794 (12.5)
Renal and urinary disorders	4,788 (75.5)
Diabetic nephropathy	1,610 (25.4)
Glomerulonephritis	696 (11.0)
Hypertensive nephropathy	1,949 (30.7)
Dialysis prior to study entry	
Yes	2,274 (35.8)
No	4,070 (64.1)
Dialysis per week, n	2,268
Mean (SD)	3.1 (0.8)
Median (range)	3.0 (0 – 7)

Values are n (%) unless stated otherwise. *Patients with Race = Other: European were counted as White. ^†^Heart rate = 0 was documented for 1 patient in each group. It was confirmed that heart rate was not assessed for both patients. ^‡^Selected risk factors observed in ≥ 5% of patients by preferred term. Group R = patients who were treated with Retacrit; group S = patients who were treated with Silapo; SD = standard deviation.

**Table 2.** Adverse events of special interest in the safety set.

AESI	Total (n = 6,337)
Number of patients with at least 1 AESI	418 (6.60)
Number of AESIs	527
Deaths due to AE*	143
PRCA	1 (0.02)
Blood and lymphatic system disorders	1 (0.02)
Aplasia pure red cell^†^	1 (0.02)
Lack of efficacy	34 (0.54)
General disorders and administration site conditions	34 (0.54)
Drug ineffective	33 (0.52)
Therapeutic product effect decreased	1 (0.02)
Thromboembolic events	389 (6.14)
Cardiac disorders	163 (2.57)
Acute myocardial infarction	47 (0.74)
Coronary artery occlusion	2 (0.03)
Coronary artery thrombosis	1 (0.02)
Intracardiac thrombus	1 (0.02)
Myocardial infarction	114 (1.80)
Eye disorders	4 (0.06)
Retinal artery occlusion	1 (0.02)
Retinal infarction	1 (0.02)
Retinal vein thrombosis	2 (0.03)
Gastrointestinal disorders	4 (0.06)
Intestinal infarction	3 (0.05)
Mesenteric artery stenosis	1 (0.02)
Mesenteric vein thrombosis	1 (0.02)
Injury, poisoning and procedural complications	41 (0.65)
Arterial bypass occlusion	1 (0.02)
Arteriovenous fistula occlusion	1 (0.02)
Arteriovenous fistula thrombosis	2 (0.03)
Carotid artery restenosis	1 (0.02)
Shunt occlusion	27 (0.43)
Shunt thrombosis	9 (0.14)
Subdural hematoma	3 (0.05)
Vascular graft occlusion	1 (0.02)
Nervous system disorders	114 (1.80)
Basal ganglia hemorrhage	2 (0.03)
Cerebellar hematoma	1 (0.02)
Cerebellar infarction	1 (0.02)
Cerebral artery occlusion	1 (0.02)
Cerebral hemorrhage	14 (0.22)
Cerebral infarction	8 (0.13)
Cerebral ischemia	4 (0.06)
Cerebrovascular accident	33 (0.52)
Cerebrovascular disorder	1 (0.02)
Embolic cerebral infarction	1 (0.02)
Embolic stroke	1 (0.02)
Hemorrhagic stroke	2 (0.03)
Hemiparesis	1 (0.02)
Ischemic stroke	34 (0.54)
Transient ischemic attack	18 (0.28)
Product issues	2 (0.03)
Thrombosis in device	2 (0.03)
Renal and urinary disorders	1 (0.02)
Renal artery thrombosis	1 (0.02)
Respiratory, thoracic, and mediastinal disorders	24 (0.38)
Pulmonary embolism	23 (0.36)
Pulmonary thrombosis	1 (0.02)
Surgical and medical procedures	1 (0.02)
Arterial stent insertion	1 (0.02)
Vascular disorders	83 (1.31)
Aortic thrombosis	1 (0.02)
Arterial occlusive disease	10 (0.16)
Arterial thrombosis	2 (0.03)
Deep vein thrombosis	13 (0.21)
Embolism	12 (0.19)
Embolism arterial	1 (0.02)
Iliac artery occlusion	1 (0.02)
Infarction	2 (0.03)
Pelvic venous thrombosis	2 (0.03)
Peripheral arterial occlusive disease	30 (0.47)
Peripheral artery occlusion	3 (0.05)
Peripheral artery thrombosis	1 (0.02)
Peripheral embolism	2 (0.03)
Subclavian vein thrombosis	2 (0.03)
Thrombophlebitis superficial	2 (0.03)
Thrombosis	5 (0.08)
Venous occlusion	1 (0.02)
Venous thrombosis	1 (0.02)

Values are n (%). AESI included PRCA, neutralizing antibodies, lack of efficacy and thromboembolic events including cerebrovascular events (e.g., cerebrovascular accident, cerebral infarction, cerebral hemorrhage, and transient ischemic attack), deep vein thrombosis, myocardial infarction, and pulmonary embolism. AESIs were summarized by type of AESI, system organ class, and preferred term. Patients were counted once within each system organ class or for each preferred term and may have had more than 1 AE. The Medical Dictionary for Regulatory Activities (MedDRA) Version 23 coding dictionary was applied. *Two additional patients (not counted in Table 2) died due to AESI after completing the study observation. ^†^“Aplasia pure red cell” is the MedDRA preferred term of “pure red cell aplasia”. AE = adverse event; AESI = adverse event of special interest; PRCA = pure red cell aplasia.

**Table 3.** Exposure-adjusted incidence rate of AEs, AESIs, and ADRs in the safety set.

	Total (n = 6,337)	
	Number of patients with AE, n (%) [95% CI]	Incidence rate (patients with AE/100 patient-years), [95% CI]
AEs	441 (6.96) [6.34, 7.61]	3.84 [3.49, 4.20]
AESIs	418 (6.60) [6.00, 7.24]	3.63 [3.30, 3.99]
PRCA	1 (0.02) [0.0008, 0.10]	0.0084 [0.0004, 0.05]
Neutralizing antibodies	1 (0.02) [0.0008, 0.10]	0.0084 [0.0004, 0.05]
Lack of efficacy	34 (0.54) [0.37, 0.75]	0.29 [0.20, 0.40]
Thromboembolic events	389 (6.14) [5.56, 6.76]	3.37 [3.05, 3.72]
ADRs other than AESIs	28 (0.44) [0.29, 0.64]	0.24 [0.16, 0.34]

AESIs included PRCA, neutralizing antibodies, lack of efficacy and thromboembolic events including cerebrovascular events (e.g., cerebrovascular accident, cerebral infarction, cerebral hemorrhage, and transient ischemic attack), deep vein thrombosis, myocardial infarction, and pulmonary embolism. ADR = adverse drug reaction; AE = adverse event; AESI = AE of special interest; CI = confidence interval; PRCA = pure red cell aplasia.

## Supplemental material

Supplemental material
