# Supplementary material for: Three-year safety observation of subcutaneous administration of epoetin-zeta in patients with chronic renal anemia: Results from PASCO II study
Source: Clin Nephrol. 2023 Mar 5;99(5):247–55. doi: 10.5414/CN110825 (PMC10112001; doi:10.5414/CN110825)
Supplement: Supplemental material [file clinnephrol-99-247-S01.pdf]

Long-term safety observation of subcutaneous administration of epoetin-zeta in patients with chronic renal anemia: results from PASCO II study

Stavros Patsialas[1], Heather Fowler[2], Ruffy Guilatco[3], Stephanie Salts[4], Feng Richard Xia[5], Sonja Gomez Perez[6], Andreas Iwanowitsch[6], Matthias Kohnle[7]

[1]*Nephroiasis Dialysis Unit, 25 Martiou 10 Efkarpia, Thessaloniki, Greece*
[2]*Pfizer Ltd, Walton Oaks, Walton-on-the-Hill, Surrey KT20 7NS, United Kingdom*
[3]*Pfizer Inc., Rockwell Center, Poblacion, Makati City, Philippines*
[4]*Pfizer Inc., 10770 Science Center Drive, San Diego, CA 92121, USA*
[5]*Pfizer Inc., 235 E 42nd St, New York, NY 10017, USA*
[6]*STADA Arzneimittel AG, Stadastraße 2-18, 61118 Bad Vilbel, Germany*
[7]*Nephrocare Mettmann GmbH, Gartenstraße 4-8, D-40822 Mettmann, Germany*

Corresponding author: Matthias Kohnle, MD, Nephrocare Mettmann GmbH, Gartenstraße 4-8, D-40822 Mettmann, Germany. Tel: +49 2104 97 99 60; Fax: +49 2104 97 99 671; Email: matthias.kohnle@nephrocare.com.

Supplementary Materials

Contents

Supplementary Table 1. Overview on the safety set 2
Supplementary Table 2. Serious AESIs in the safety set 3
Supplementary Table 3. ADRs other than AESIs by system organ class and preferred term in the safety set 5
Supplementary Table 4. Exposure to epoetin-zeta in patient-years by duration in the safety set 6

Supplementary Table 1. Overview on the safety set

	**Total (n=6,337)**
Number of AEs	568
Patients discontinued from study due to AEs	244 (3.9)
Patients died due to AEs	143 (2.3)
Patients with AEs	441 (7.0)
Patients with serious AEs	415 (6.6)
Patients with AESI	418 (6.6)
Patients with serious AESI	409 (6.5)
Patients with ADRs other than AESI	28 (0.4)
Patients with serious ADRs other than AESI	11 (0.2)

Values are n (%).

AESIs included PRCA, neutralizing antibodies, lack of efficacy and thromboembolic events including cerebrovascular events (e.g., cerebrovascular accident, cerebral infarction, cerebral haemorrhage, and transient ischaemic attack), deep vein thrombosis, myocardial infarction, and pulmonary embolism.

ADR, adverse drug reactions; AE, adverse event; AESI, AEs of special interest; PRCA, pure red cell aplasia.

Supplementary Table 2. Serious AESIs in the safety set

**AESI**	**Total (n=6,337)**
Number of patients with at least one serious AESI	409 (6.45)
Number of serious AESIs	516
PRCA	1 (0.02)
Blood and lymphatic system disorders	1 (0.02)
Aplasia pure red cell*	1 (0.02)
Lack of Efficacy	26 (0.41)
General disorders and administration site conditions	26 (0.41)
Drug ineffective	25 (0.39)
Therapeutic product effect decreased	1 (0.02)
Thromboembolic events	387 (6.11)
Cardiac disorders	163 (2.57)
Acute myocardial infarction	47 (0.74)
Coronary artery occlusion	2 (0.03)
Coronary artery thrombosis	1 (0.02)
Intracardiac thrombus	1 (0.02)
Myocardial infarction	114 (1.80)
Eye disorders	4 (0.06)
Retinal artery occlusion	1 (0.02)
Retinal infarction	1 (0.02)
Retinal vein thrombosis	2 (0.03)
Gastrointestinal disorders	4 (0.06)
Intestinal infarction	3 (0.05)
Mesenteric artery stenosis	1 (0.02)
Mesenteric vein thrombosis	1 (0.02)
Injury, poisoning and procedural complications	41 (0.65)
Arterial bypass occlusion	1 (0.02)
Arteriovenous fistula occlusion	1 (0.02)
Arteriovenous fistula thrombosis	2 (0.03)
Carotid artery restenosis	1 (0.02)
Shunt occlusion	27 (0.43)
Shunt thrombosis	9 (0.14)
Subdural haematoma	3 (0.05)
Vascular graft occlusion	1 (0.02)
Nervous system disorders	112 (1.77)
Basal ganglia haemorrhage	2 (0.03)
Cerebellar haematoma	1 (0.02)
Cerebellar infarction	1 (0.02)
Cerebral artery occlusion	1 (0.02)
Cerebral haemorrhage	14 (0.22)
Cerebral infarction	7 (0.11)
Cerebral ischaemia	4 (0.06)
Cerebrovascular accident	33 (0.52)
Cerebrovascular disorder	1 (0.02)
Embolic cerebral infarction	1 (0.02)
Embolic stroke	1 (0.02)
Haemorrhagic stroke	2 (0.03)

Hemiparesis	1 (0.02)
Ischaemic stroke	34 (0.54)
Transient ischaemic attack	17 (0.27)
Product issues	2 (0.03)
Thrombosis in device	2 (0.03)
Renal and urinary disorders	1 (0.02)
Renal artery thrombosis	1 (0.02)
Respiratory, thoracic and mediastinal disorders	24 (0.38)
Pulmonary embolism	23 (0.36)
Pulmonary thrombosis	1 (0.02)
Surgical and medical procedures	1 (0.02)
Arterial stent insertion	1 (0.02)
Vascular disorders	82 (1.29)
Aortic thrombosis	1 (0.02)
Arterial occlusive disease	10 (0.16)
Arterial thrombosis	2 (0.03)
Deep vein thrombosis	13 (0.21)
Embolism	11 (0.17)
Embolism arterial	1 (0.02)
Iliac artery occlusion	1 (0.02)
Infarction	2 (0.03)
Pelvic venous thrombosis	2 (0.03)
Peripheral arterial occlusive disease	30 (0.47)
Peripheral artery occlusion	3 (0.05)
Peripheral artery thrombosis	1 (0.02)
Peripheral embolism	2 (0.03)
Subclavian vein thrombosis	2 (0.03)
Thrombophlebitis superficial	2 (0.03)
Thrombosis	5 (0.08)
Venous occlusion	1 (0.02)
Venous thrombosis	1 (0.02)

Values are n (%). AESIs were summarised by type of AESI, system organ class and preferred term. Patients were counted once within each system organ class or for each preferred term and may have had more than 1 AE. The Medical Dictionary for Regulatory Activities (MedDRA) Version 23 coding dictionary was applied.

AESIs included PRCA, neutralizing antibodies, lack of efficacy and thromboembolic events including cerebrovascular events (e.g., cerebrovascular accident, cerebral infarction, cerebral haemorrhage, and transient ischaemic attack), deep vein thrombosis, myocardial infarction, and pulmonary embolism.

*The MedDRA preferred term of “pure red cell aplasia” is “aplasia pure red cell”.

AESI, adverse event of special interest; PRCA, pure red cell aplasia.

Supplementary Table 3. ADRs other than AESIs by system organ class and preferred term in the safety set

**ADR**	**Total (n=6,337)**
Number of patients with at least one ADR other than AESI	28 (0.44)
Number of ADRs other than AESI	41
Cardiac disorders	2 (0.03)
Arrhythmia	1 (0.02)
Palpitations	1 (0.02)
Gastrointestinal disorders	4 (0.06)
Diarrhoea	1 (0.02)
Lip swelling	1 (0.02)
Nausea	2 (0.03)
Swollen tongue	1 (0.02)
Vomiting	1 (0.02)
General disorders and administration site conditions	4 (0.06)
Influenza like illness	1 (0.02)
Malaise	3 (0.05)
Infections and infestations	1 (0.02)
Gangrene	1 (0.02)
Investigations	5 (0.08)
Haemoglobin decreased	5 (0.08)
Musculoskeletal and connective tissue disorders	1 (0.02)
Bone pain	1 (0.02)
Nervous system disorders	5 (0.08)
Dizziness	2 (0.03)
Dyskinesia	1 (0.02)
Headache	3 (0.05)
Somnolence	1 (0.02)
Psychiatric disorders	1 (0.02)
Nightmare	1 (0.02)
Reproductive system and breast disorders	2 (0.03)
Breast disorder	1 (0.02)
Vulvovaginal pruritus	1 (0.02)
Skin and subcutaneous tissue disorders	9 (0.14)
Angioedema	1 (0.02)
Dermatitis allergic	2 (0.03)
Dermatitis atopic	1 (0.02)
Eczema	1 (0.02)
Hypertrichosis	1 (0.02)
Pruritus	3 (0.05)
Rash	1 (0.02)
Vascular disorders	2 (0.03)
Hypertension	1 (0.02)
Hypertensive urgency	1 (0.02)

Values are n (%).

ADR, adverse drug reaction; AESI, adverse events of special interest.

Supplementary Table 4. Exposure to epoetin-zeta in patient-years by duration in the safety set

	**Total (n=6,337)**	
Duration of treatment	n (%)	Patient-years
Cumulative up to 6 months	991 (15.6)	253.3
Cumulative up to 12 months	1,859 (29.3)	955.0
Cumulative up to 18 months	2,644 (41.7)	1,993.7
Cumulative up to 24 months	3,342 (52.7)	3,264.5
Cumulative up to 30 months	3,830 (60.4)	4,394.1
Cumulative up to 36 months	5,322 (84.0)	8,703.0
Cumulative up to and beyond 36 months	6,337 (100.0)	11,837.6
